# Retinoic acid exerts sexually dimorphic effects on muscle energy metabolism and function

**DOI:** 10.1016/j.jbc.2021.101101

**Published:** 2021-08-19

**Authors:** Yaxin Zhao, Marta Vuckovic, Hong Sik Yoo, Nina Fox, Adrienne Rodriguez, Kyler McKessy, Joseph L. Napoli

**Affiliations:** Graduate Program in Metabolic Biology, Department of Nutritional Sciences and Toxicology, The University of California-Berkeley, Berkeley, California, USA

**Keywords:** ATP, cytochrome c oxidase (complex IV), estrogen, gene knockout, metabolic regulation, Rdh10, retinoic acid, running endurance, skeletal muscle, vitamin A, CLAMS, Comprehensive laboratory animal monitoring system, CSA, cross-sectional areas, GM, gastrocnemius muscle, Het, *Rdh10+/−*, HFD, high-fat diet, RA, all-*trans*-retinoic acid, RER, respiratory exchange ratio, TG, triacylglycerol, WT, wild type

## Abstract

The retinol dehydrogenase Rdh10 catalyzes the rate-limiting reaction that converts retinol into retinoic acid (RA), an autacoid that regulates energy balance and reduces adiposity. Skeletal muscle contributes to preventing adiposity, by consuming nearly half the energy of a typical human. We report sexually dimorphic differences in energy metabolism and muscle function in *Rdh10*+/− mice. Relative to wild-type (WT) controls, *Rdh10*+/− males fed a high-fat diet decrease reliance on fatty-acid oxidation and experience glucose intolerance and insulin resistance. Running endurance decreases 40%. *Rdh10*+/− females fed this diet increase fatty acid oxidation and experience neither glucose intolerance nor insulin resistance. Running endurance increases 220%. We therefore assessed RA function in the mixed-fiber type gastrocnemius muscles (GM), which contribute to running, rather than standing, and are similar to human GM. RA levels in *Rdh10*+/− male GM decrease 38% relative to WT. *Rdh10*+/− male GM increase expression of *Myog* and reduce *Eif6* mRNAs, which reduce and enhance running endurance, respectively. Cox5A, complex IV activity, and ATP decrease. Increased centralized nuclei reveal existence of muscle malady and/or repair in GM fibers. Comparatively, RA in *Rdh10*+/− female GM decreases by less than half the male decrease, from a more modest decrease in *Rdh10* and an increase in the estrogen-induced retinol dehydrogenase *Dhrs9*. *Myog* mRNA decreases. Cox5A, complex IV activity, and ATP increase. Centralized GM nuclei do not increase. We conclude that Rdh10/RA affects whole body energy use and insulin resistance partially through sexual dimorphic effects on skeletal muscle gene expression, structure, and mitochondria activity.

All-*trans*-retinoic acid (RA) regulates embryonic development, cell proliferation, differentiation, and numerous functions of differentiated cells through complex genomic and nongenomic mechanisms (1, 2, 3, 4). RA biogenesis stems from either retinol (vitamin A) or β-carotene (5, 6, 7, 8). As many as eight retinol dehydrogenases and reductases, of the short-chain dehydrogenase/reductase gene family, catalyze conversion of retinol into retinal, and retinal into retinol (9). At least three retinal dehydrogenases, of the aldehyde dehydrogenase gene family, convert retinal generated from retinol or β-carotene, irreversibly into RA. Cells coexpress multiple retinoid metabolic enzymes, but gene ablations reveal dissimilar phenotypes for each, consistent with distinct contributions to RA function (10, 11). For example, knocking out the retinol dehydrogenase *Rdh1* increases adiposity in mice fed a low-fat diet, from decreased RA in brown adipocytes, which impairs lipolysis and fatty acid oxidation (12). In contrast, the retinol dehydrogenase *Dhrs9* seems crucial to the hair follicle cycle, and its decreased expression correlates with squamous cell carcinoma (13, 14). Ablation of the retinol dehydrogenase *Rdh10* causes lethality between embryo days 10.5 to 14.5 from impaired nervous system and craniofacial development (15, 16, 17). During postnatal growth, Rdh10 generates RA to support spermatogenesis and organ repair and to regulate energy use (18, 19, 20). Ablation of the retinal reductase *Dhrs3* increases embryonic RA amounts by ∼40% and causes death late in gestation from defects in heart development (21, 22). Consistent with partially overlapping contributions to RA homeostasis, reducing expression of one reductase/dehydrogenase can provoke compensation by others. These observations prompt questions about the needs for and functions of so many enzymes dedicated to RA homeostasis.

Establishing precise physiological functions of endogenous RA cannot be attained by feeding vitamin A-deficient diets or pharmacological RA dosing, which prompts RA toxicity. The heterozygote *Rdh10*-null mouse (*Rdh10+/−*) experiences a limited decrease in tissue RA (≤25%), allowing reproducible assessment of endogenous RA functions *in vivo* (20). When fed a high-fat (western) diet, *Rdh10+/−* mice gain adipose and males suffer liver steatosis, whereas females form adipocytes in bone. To develop further insight into the function of Rdh10 and physiological RA actions, we expanded evaluation of the *Rdh10+/−* mouse to include whole-body energy metabolism and skeletal muscle function. Skeletal muscle accounts for about half of all energy used, producing not only work but also heat to defend body temperature (23). Exercise, through muscles’ bountiful demand for energy, ameliorates obesity and insulin resistance (24, 25). Energy metabolism, obesity, metabolic syndrome, and muscle function interweave to affect overall health.

RA induces skeletal muscle development through controlling expression of myogenic regulatory factors, including Myf5, MyoD, and Myog during embryogenesis and in primary myoblasts and cell lines (26, 27, 28, 29). RA induces proliferation of cultured myoblasts and sustains their survival, suggesting a contribution to skeletal muscle regeneration (30, 31). Pharmacological dosing with RARγ agonists stimulates skeletal muscle repair and reduces extent of fatty fibrotic tissue lesions in a muscle injury model (32). Although it exerts crucial actions during muscle development and repair, little has been revealed about RA function on muscle performance *in vivo*.

Here we report sexually dimorphic differences in *Rdh10+/−* mice fed a high-fat diet (HFD) in the respiratory exchange ratio (RER), running endurance, glucose tolerance, and insulin resistance. Decreased Rdh10 in male gastrocnemius muscle (GM), a mixed-fiber type muscle, associates with TAG accumulation, an increase in centralized nuclei, reduced expression and activity of complex IV components of the electron transport chain, and decreased ATP. *Rdh10+/−* females have increased complex IV components and activity and a higher concentration of resting ATP than WT. Estrogen increased in *Rdh10+/−* females, as did expression of the estrogen-induced retinol dehydrogenase *Dhrs9* (33). RA decreased in *Rdh10+/−* female GM less than half the male decrease, likely due to compensation by Dhrs9. These results provide new insight into the physiological functions of Rdh10, sex differences in RA biosynthesis, and actions related to energy use, insulin resistance, and muscle function.

## Results

### *Rdh10+/−* differ by sex from WT in fuel use

**Figure 1:**
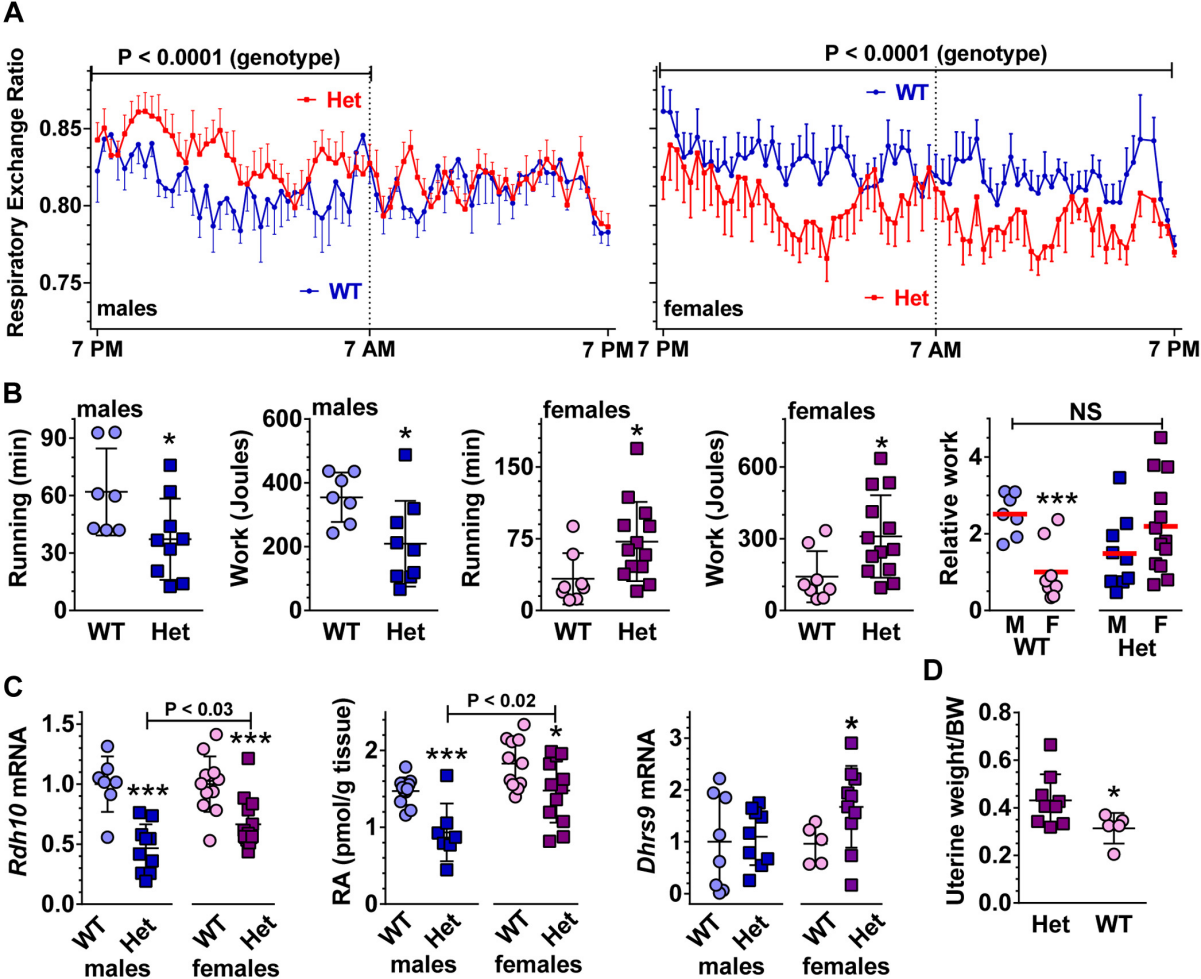
***Rdh10+/−* (Het) males and females differ from each other and WT in GM RA, fuel use, running endurance, and myogenic transcription factor expression.***A*, respiratory exchange ratio (CO_2_/O_2_) during ambient temperature and ad lib feeding a high-fat diet (HFD). Data are means ± SEM (n = 6–10 mice/genotype/sex). Data were analyzed by ordinary two-way ANOVA from 75 equally spaced observations over 24 h for each sex/genotype to assess genotypic differences. *B*, running time and work done during the run-to-exhaustion test (n = 7–13). The *last panel* shows work done relative to female WT average of 1. *Red bars* denote averages. *C*, *Rdh10* mRNA (n = 7–14), RA content (n = 12), *Dhrs9* mRNA (n = 7–13) in GM. *D*, weight of uteri relative to total body weight (BW) (n = 5–9). Symbols in all seven figures: WT male, *light blue circles*; *Rdh10+/−* (Het) male, *dark blue squares*; WT female, *light pink circles*; *Rdh10+/−* (Het) female, *dark purple squares*.

*Rdh10+/−* males fed a purified HFD from weaning endure glucose intolerance and insulin resistance, but females were not assessed (20). Here we found that *Rdh10+/−* females of the same age and fed a purified HFD diet with the same formulation since weaning did not experience glucose intolerance or insulin resistance (Figs. S1 and S2). Further, the RER (CO_2_ exhaled/O_2_ consumed) showed that WT males relied more on fatty acid oxidation than WT females during normal activity (Fig. 1*A*). *Rdh10+/−* males decreased fatty acid oxidation and increased carbohydrate use during the 12-h dark (feeding) cycle, relative to WT males, but did not differ from WT males during the light cycle. *Rdh10+/−* females decreased carbohydrate use and increased fat oxidation throughout 24 h, relative to WT females. RER data also are shown as averages (Fig. S3).

### *Rdh10+/−* males and females differ in running endurance

Differences in reliance on carbohydrates *versus* fatty acid oxidation implied possible endurance differences. In run-to-exhaustion tests of endurance, WT males ran an average 62 min, whereas *Rdh10+/−* males ran an average 37 min (40% decrease), resulting in an equivalent 40% decrease in work accomplished (Fig. 1*B*). WT females ran an average 33 min, *i.e.*, ∼47% less than WT males. In contrast, *Rdh10+/−* females ran an average 72 min, ∼2.2-fold longer than WT females with the same % increase in work performed. Thus, loss of one *Rdh10* copy reversed the relative running endurance of males *versus* females.

### *Rdh10+/−* males and females differ in GM RA concentrations

To examine mechanisms, we focused on GM, because they perform predominantly in running, rather than standing, and have the most extensive overlap of pathways in mouse *versus* human (34). *Rdh10* mRNA in GM in *Rdh10+/−* mice declined by 53% and 33% in Het males and females, respectively (Fig. 1*C*). RA decreased 38% and 17% in GM of *Rdh10+/−* males and females, respectively. Estrogen induces the retinol dehydrogenase *Dhrs9* (33). *Dhrs9* mRNA in male *Rdh10+/−* mice did not differ significantly from WT, whereas *Dhrs9* mRNA in female *Rdh10+/−* mice exceeded WT by 1.7-fold. Other mRNAs of the retinoid metabolon, including the retinal dehydrogenases *Raldh1*, *2*, *3*, and the RA catabolic enzyme *Cyp26b1*, were unaffected in both sexes, as was the putative retinol dehydrogenase *Dhr7c* (Fig. S4). The combined sex-specific differences in *Rdh10* and *Dhrs9* mRNAs would contribute to the difference between RA concentrations in *Rdh10+/−* males *versus* females.

Because estrogen induces *Dhrs9*, we attempted to quantify estrogen concentrations in female sera. We were unable to detect estrogen in sera of female mice using an LC/MS/MS assay with a 2 fmol lower limit of detection (35). Estrogen concentrations in mice sera occur below limits of detection, unless females are exposed to males, because group-housed female mice enter anestrus. Instead, we quantified uterine weight, which reflects estrogen levels (36, 37). *Rdh10+/−* uteri weighed 39% more than WT relative to total body weight, reflecting an estrogen increase (Fig. 1*D*). Because of these differential sex differences in response to *Rdh10* knockdown, data from each sex were compared with their own littermate WT mice.

### *Rdh10+/−* males and females differ in myogenic transcription factor expression

**Figure 2:**
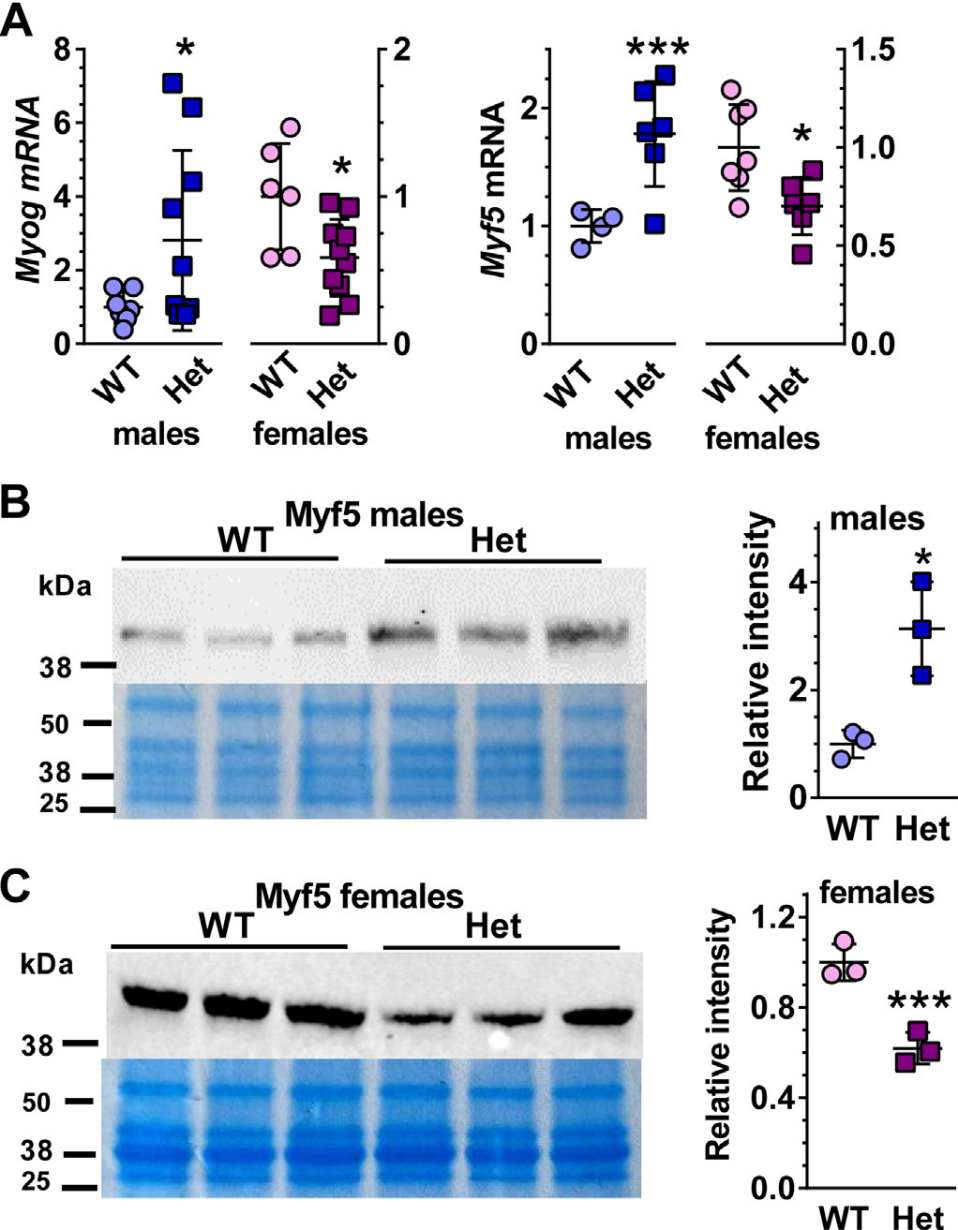
***Rdh10+/−* (Het) males and females differ from each other and from WT in myogenic transcription factor expression.***A*, mRNA of myogenic transcription factors *Myog* (n = 6–12) and *Myh5* (n = 4–12) in GM. *B* and *C*, western blots of Myf5 in male and female GM, respectively. Data expressed as relative to WT (=1) for each sex. Coomassie blue images were reused in Figures 5, *B* and *C* and 7*B*.

mRNA levels of the RA-regulated myogenic transcription factor *Myog* (myogenin) increased 2.8-fold in *Rdh10+/−* male GM, relative to WT (Fig. 2*A*). In contrast, *Rdh10+/−* female *Myog* mRNA decreased by 42%, relative to WT. mRNA of another RA-regulated myogenic transcription factor, *Myf5*, also increased in *Rdh10+/−* males (1.8-fold), whereas it decreased 30% in females. The mRNA of a third myogenic transcription factor *Myod* did not differ between WT and *Rdh10+/−* GM of either sex (Fig. S5). To verify that mRNA levels represented protein levels, we performed western blots on Myf5 (Fig. 2, *B* and *C*). The data reflected adaptations in the mRNA. *Rdh10+/−* male Myf5 increased threefold, whereas *Rdh10+/−* female Myf5 decreased 38%. Other factors that regulate skeletal muscle mass and function include myostatin (Mstn), atrogin-1, and MuRF-1 (38, 39, 40). Mice deficient in Mstn have marked increases in skeletal muscle mass, because Mstn suppresses myogenesis by interfering with Myod. Striated muscle expresses atrogin-1 specifically. Atrogin-1 and MuRF are ubiquitin ligases that steer muscle protein to proteolysis. *Mstn* and *Murf1* mRNA did not change in *Rdh10+/−* of either sex relative to WT. The lack of differences in *Myod* in *Rdh10+/− versus* WT reflects the lack of differences in *Mstn* mRNA. *Atrogin-1* mRNA did not change in *Rdh10+/−* males relative to WT, but decreased 23% in *Rdh10+/−* females (Fig. S6). Other genes associated with enhanced or diminished endurance in mice did not change, including *Adcy5*, *Adrb2*, *Hif1a*, *Ppard*, *Pten*, *Scd1*, and *Uchl1* (Fig. S7) (41).

### Rdh10 affects GM fiber types in males

**Figure 3:**
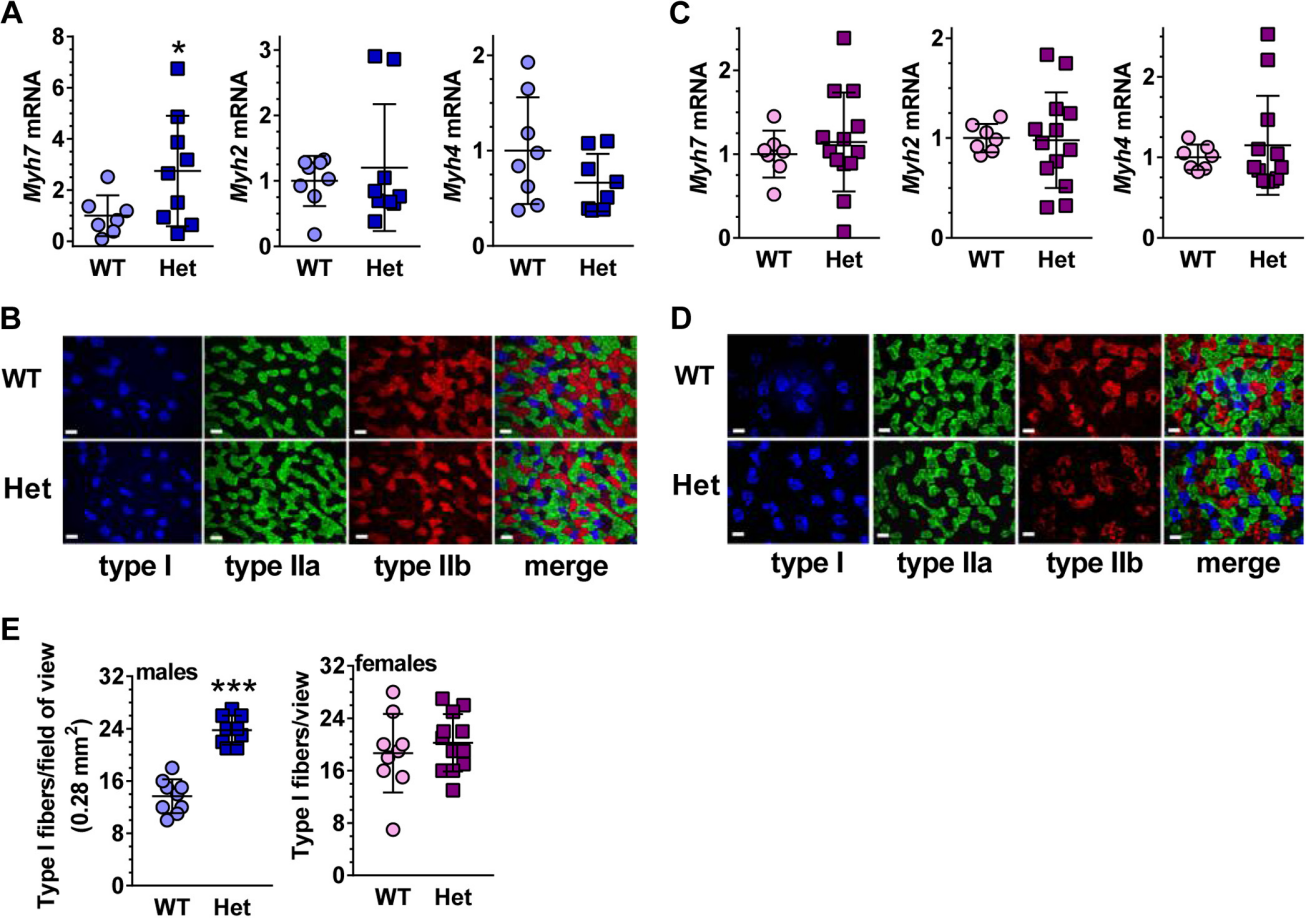
**Rdh10 constrains the nature of GM fibers.***A*, mRNA of male *Myh7* (type I), *Myh2* (type IIa), and *Myh4* (IIb) genes (n = 7–9). *B*, immunofluorescence of male GM fiber types. *C*, mRNA of female *Myh7*, *Myh2* and *Myh4* genes (n = 7–13). *D*, immunofluorescence of female GM fiber types. *E*, quantification of type I fibers (n = 9). Bars = 50 μm. Het, *Rdh10+/−*.

Differential myosin heavy chain (*Myh*) expression designates skeletal muscle fibers as type I (*Myh7*), type IIa (*Myh2*), or type IIb (*Myh4*) (42). Type I (slow-twitch) fibers have abundant mitochondria and promote endurance. These fibers rely mainly on fatty acid oxidation. Type IIa fibers rely on both oxidative and glycolytic pathways. Type IIb fibers (fast-twitch or “sprint”) have few mitochondria and rely mainly on glycolysis. GM consist of mixed fiber types, with ∼54% type IIb fibers, and the rest distributed among types I and IIa/d. The mRNA of *Myh7*, an indicator of type I fibers, increased approximately threefold in *Rdh10+/−* male GM (Fig. 3, *A* and *B*). No changes occurred in *Rdh10+/− Myh2* (type IIa fibers) or *Myh4* (IIb fibers) mRNA in male GM. *Rdh10+/−* female GM *Myh7* mRNA remained the same as WT, as did *Myh2* and *Myh4* (Fig. 3, *C* and *D*). Quantification of fiber numbers verified a 1.7-fold increase in *Rdh10+/−* male type I fibers and no change in female type I fibers (Fig. 3*E*). Thus, *Rdh10+/−* males had decreased endurance despite compensation by increased slow-twitch oxidative fibers. This ineffective compensation by increasing slow-twitch fibers would not correct the phenotype, because the defective mitochondria in these fibers would not improve fuel use and energy production.

### *Rdh10+/−* males and females differ from each other and WT in muscle fiber characteristics

**Figure 4:**
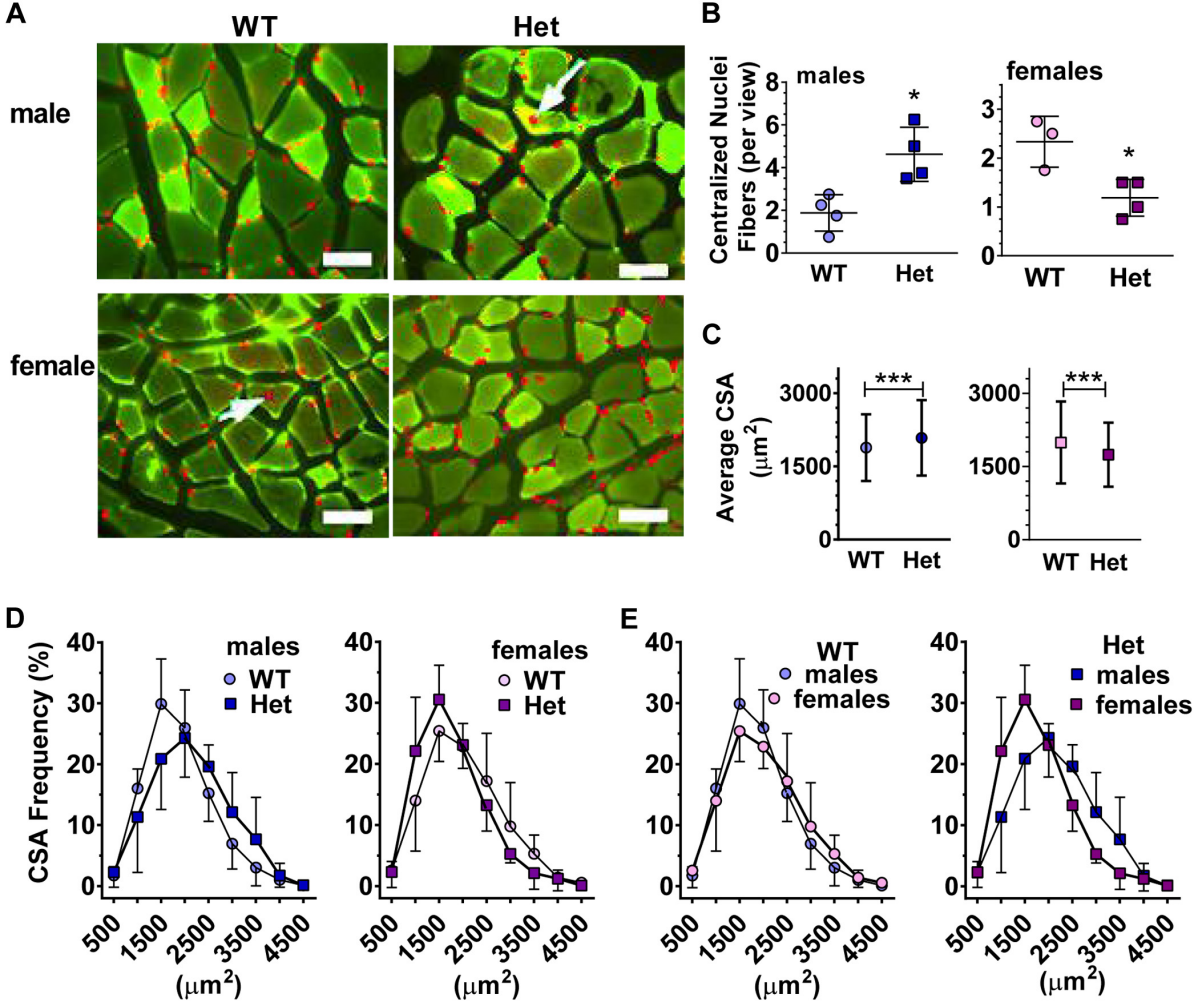
***Rdh10+/−* (Het) males and females differ from each other and WT in muscle fiber characteristics.***A*, desmin immunostaining combined with DAPI staining of GM cryosections. The *arrows* denote centralized nuclei. Bars = 50 μm. Images were overexposed to allow visualization of nuclei. *B*, quantification of average number of fibers with centralized nuclei per field of view using H&E staining (averages of 3–48 sections from each of 3–4 mice), ∗*p* < 0.02. *C*, average CSA/mouse (1454–1635 individual fibers quantified for each sex and genotype). *D* and *E*, the ranges of the CSA frequency distribution of data in *C*.

Normally, nuclei in muscle localize peripherally to fibers. Nuclei migration to the centers of fibers accompanies muscle dysfunction or regeneration (43). Figure 4*A* illustrates the difference between peripheral and centralized nuclei. Centralized nuclei increased 2.4-fold in *Rdh10+/−* male GM, but decreased 26% in *Rdh10+/−* female GM (Fig. 4*B*). Cross-sectional areas (CSA) of *Rdh10+/−* GM fibers adapted to *Rdh10* knockdown according to sex (Fig. 4*C*). Numbers of larger male fibers increased ∼10.7%, whereas numbers of larger female fibers decreased ∼12.6%. The range of Het male CSA shifted to larger ones relative to WT; the range of Het female CSA shifted to smaller ones (Fig. 4*D*). Direct comparison of male and female WT CSA revealed extensive similarity, indicating CSA normally do not diverge substantially according to sex (Fig. 4*E*). All four curves (WT and *Rdh10+/−*, male and female) had similar if not identical areas, consistent with shifts in fiber size distributions in *Rdh10+/−*, rather than muscle growth or reduction. Although *Rdh10+/−* female GM did not change in fiber type, nor show an increase in centralized nuclei, homogenization for the same length of time disrupted both *Rdh10+/−* male and female GM much faster than WT (Fig. S8). This indicates that the connective tissue surrounding the muscle fibers is weaker in *Rdh10+/−* mice, suggesting impaired tissue integrity.

### *Rdh10+/−* males experience disturbances in GM lipid metabolism

**Figure 5:**
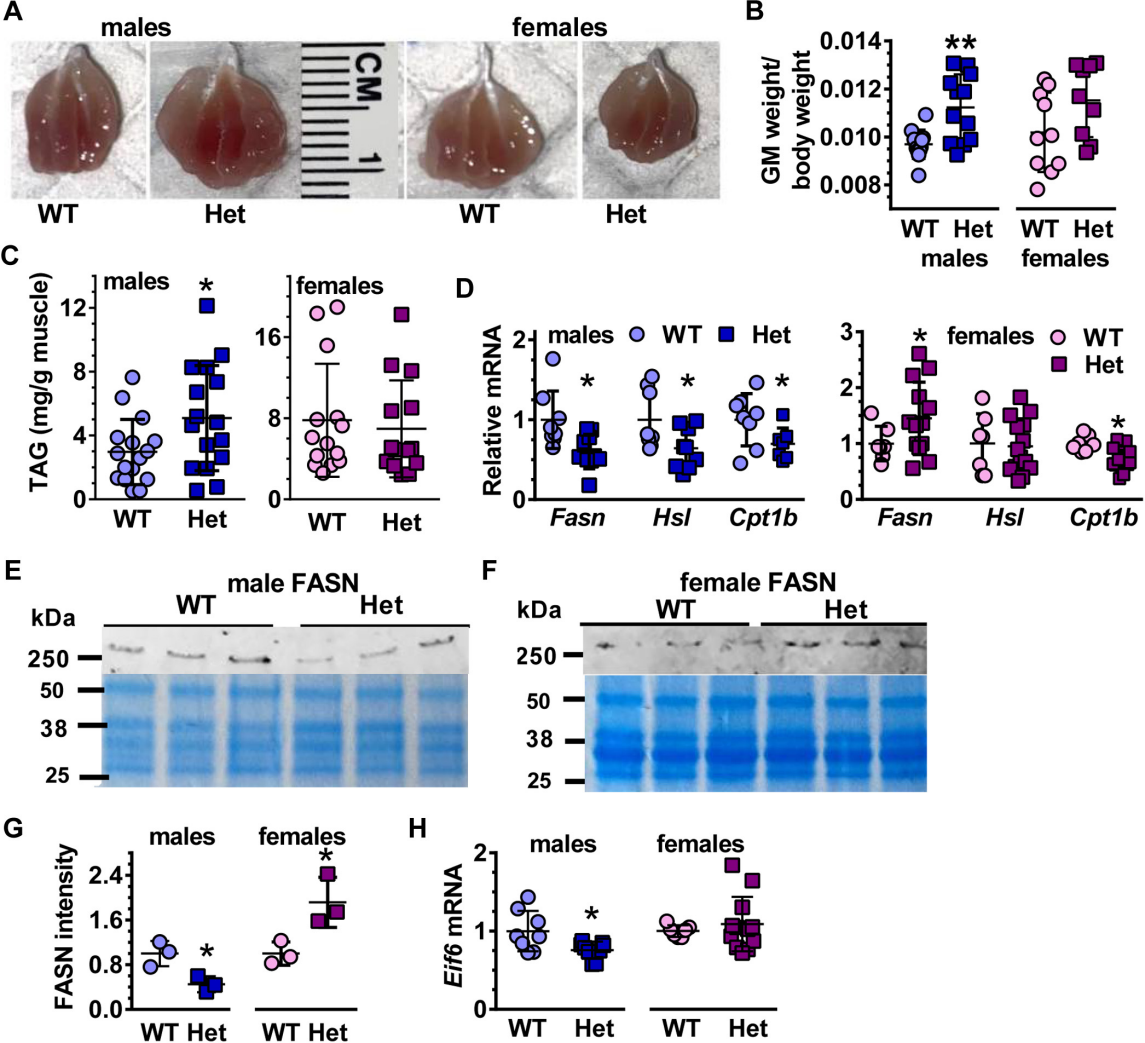
***Rdh10+/−* (Het) males experience disturbances in GM lipid metabolism.***A*, GM images. *B*, quantification of GM weights relative to total body weights (n = 12). *C*, TAG content of GM (n = 14–16). *D*, mRNA of fatty acid metabolism related genes in male GM (n = 7–10) and female GM (n = 7–14). *E*, western blots of FASN in male GM. *F*, western blots of FASN in female GM. Coomassie blue images reused from Figure 2, *B* and *C*. *G*, quantification of FASN western blots in GM. Data were normalized to the male WT signals set to 1. *H*, mRNA of *Eif6* in GM (n = 7–12).

Muscle weights relative to whole body weights also exhibited sexually dimorphic differences. *Rdh10+/−* male GM weighed 10% more than WT, whereas *Rdh10+/−* female GM weights did not differ significantly from WT (Fig. 5, *A* and *B*). The weight increase of *Rdh10+/−* males was not caused by an increase in protein content, as no marked differences occurred among GM protein amounts in either sex (Fig. S9). Oil red O staining indicated greater TAG in *Rdh10+/−* males than other groups. This was confirmed by quantification of TAG by biochemical assay, which revealed a 1.7-fold increase in *Rdh10+/−* male GM, but no increase in *Rdh10+/−* female GM relative to WT (Fig. 5*C*). The decrease in *Rdh10* triggered changes in mRNA of genes related to fatty acid biosynthesis, lipolysis, and mitochondria long-chain fatty acid import. Fatty acid synthase (*Fasn*), hormone sensitive lipase (*Hsl*), and carnitine palmitoyltransferase 1b (*Cpt1b*) mRNA decreased 30 to 40% in *Rdh10+/−* male GM (Fig. 5*D*). *Rdh10+/−* female GM had a 50% increase in *Fasn* mRNA and a 36% decrease in *Cpt1b* mRNA, with no change in *Hsl* mRNA (Fig. 5*D*). FASN protein reflected changes in mRNA, showing a 55% decrease in *Rdh10+/−* males and a 1.9-fold increase in *Rdh10+/−* females (Fig. 5, *E*–*G*). *Rdh10+/−* male and female GM mRNA did not change for *Atgl*, *CD36*, *Hk2*, *Mcad*, *Pgcla*, and *Ppara* (Fig. S10). Insulin induces the elongation and initiation factor *Eif6*, which stimulates lipogenesis in the liver and adipose, including transcription of *Fasn* (44). *Eif6*^*+/−*^ mice also endure reduced exercise endurance (34). *Eif6* mRNA decreased 26% in *Rdh10+/−* male GM, with no decrease in *Rdh10+/−* females, consistent with Het male insulin resistance, the decrease in FASN, and the decline in running endurance (Fig. 5*H*).

### Glycogen and glucose adaptations differ sexually in *Rdh10+/−* GM

**Figure 6:**
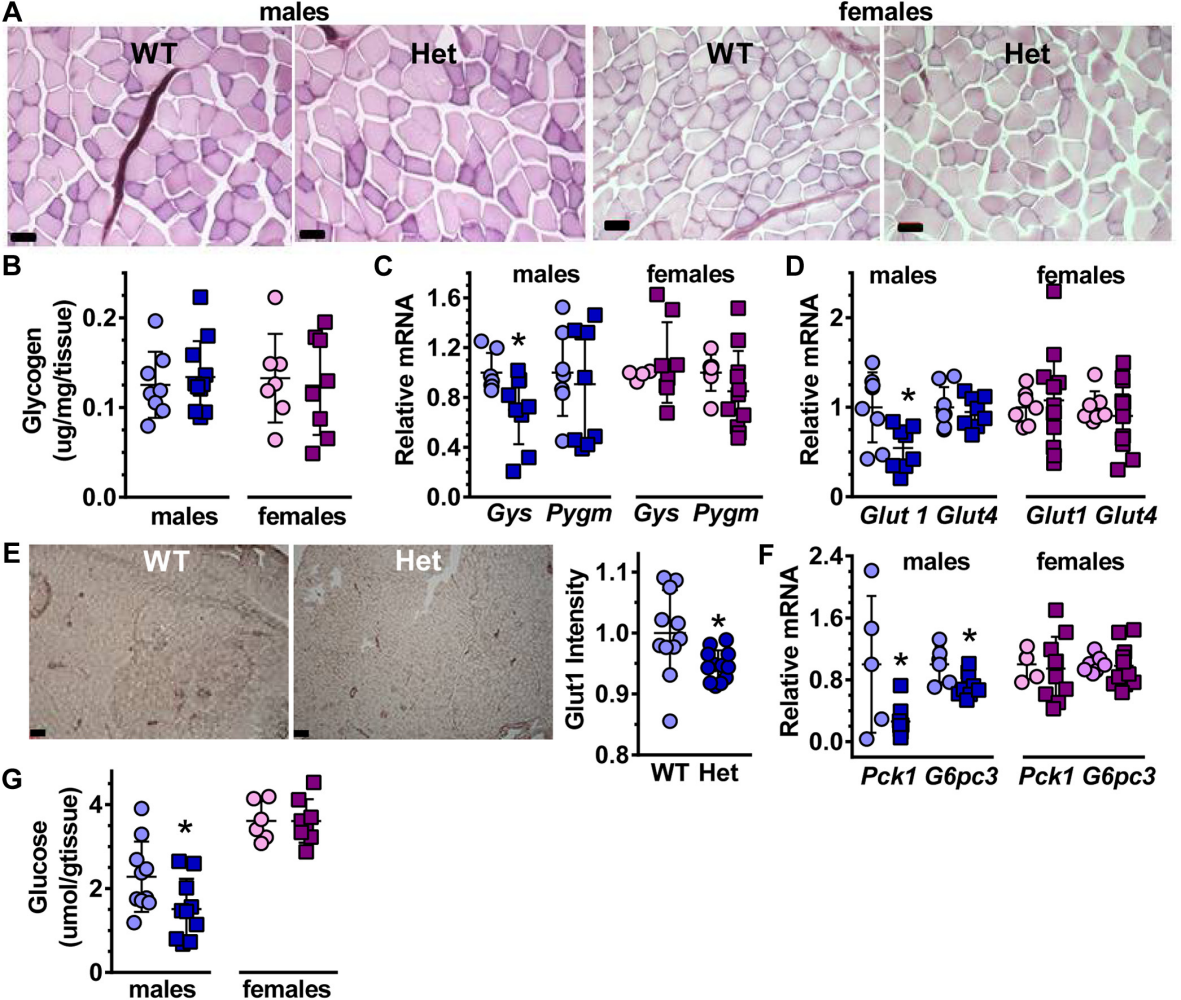
**GM Glycogen and glucose adaptations by sex in *Rdh10+/−* (Het).***A*, glycogen content (PAS staining, bars 50 μm). *B*, quantification of *A* (n = 7–11). *C*, *Gys* and *Pygm* mRNA (n = 7–11). *D*, GM *Glut1* and *Glut4* mRNA (n = 7–13). *E*, Glut1 immunostaining in males (bars 200 μm). Quantification was done on four fields of view for each of three mice/genotype. *F*, *Pck1* and *G6pc3* mRNA (n = 5–10). *G*, glucose content (n = 6–8).

The fasting glycogen content of *Rdh10+/−* GM did not differ from WT in either sex (Fig. 6, *A* and *B*). *Gys* mRNA, which encodes the rate-limiting enzyme in glycogen synthesis, decreased 30% in *Rdh10+/−* males, but did not change in females (Fig. 6*C*). *Pygm*, which encodes the rate-limiting enzyme for muscle glycogenolysis, did not change in *Rdh10+/−* mice of either sex. The lack of a decrease in muscle glycogen despite the decrease in *Gys* likely reflects 16 h fasting, which would deplete muscle glycogen.

Skeletal muscle relies for glucose uptake on glucose transporters Glut1 and Glut4. *Glut1* mRNA decreased 36% in *Rdh10+/−* male GM, but did not change in *Rdh10+/−* female GM (Fig. 6*D*). *Glut4* mRNA did not change in *Rdh10+/−* mice of either sex. The intensity of Glut1 immunostaining decreased a modest 5%, which could have a profound influence on glucose uptake, because the mice were fasted 16 h (Fig. 6*E*). The mRNA of two key genes that encode enzymes essential to gluconeogenesis, *Pck1* (phosphoenolpyruvate carboxykinase 1) and *G6pc3* (glucose-6-phosphatase catalytic subunit 3) decreased by 73 and 27%, respectively, in male GM, but did not change in female GM (Fig. 6*F*). The average amount of glucose in fasted male GM decreased by 34%, reflecting the decrease in *Glut1* and gluconeogenesis genes and the glucose intolerance and insulin resistance of male *Rdh10+/−* mice (Fig. 6*G*).

### *Rdh10+/−* males suffer reduced GM mitochondria function; females gain function

**Figure 7:**
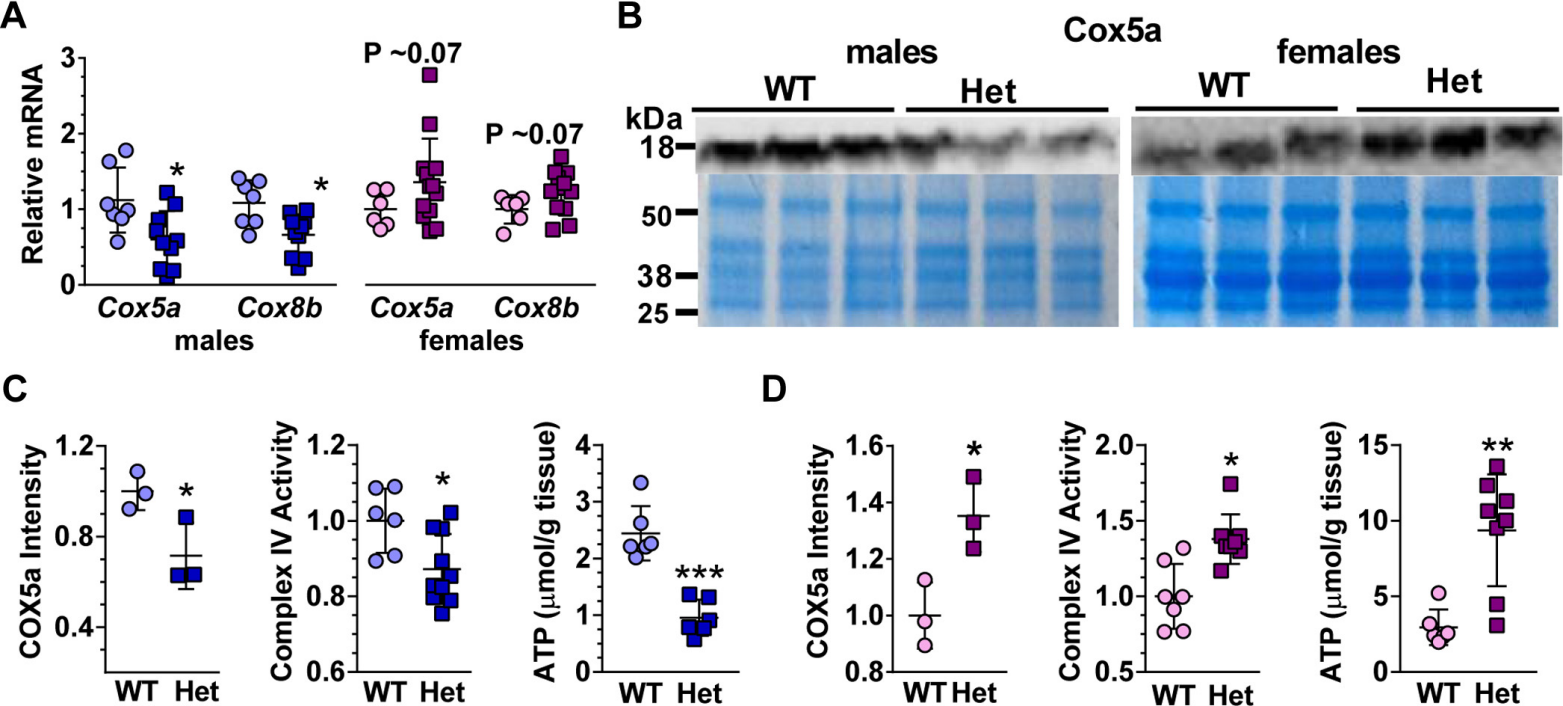
***Rdh10+/−* (Het) male GM experience reduced mitochondria function; females experience improved mitochondria function.***A*, electron transport chain complex constituents *Cox5a* and *Cox8b* mRNA in GM (n = 6–13). *B*, western blots of Cox5A in GM. Coomassie blue images were reused from Figure 2, *B* and *C*. *C*, quantification of male GM data in *B* relative to WT, relative complex IV activity (n = 6–10), and ATP concentration (n = 6 each genotype). *D*, quantification of female GM data in *B* relative to WT, relative complex 4 activity (n = 7–8), and ATP concentration (n = 6–8).

We assessed mRNA of genes associated with mitochondria function to determine the extent of electron transport chain activity. mRNAs of complex IV components (cytochrome C oxidase) *Cox5a* and *Cox8b* declined by 46% and 39%, respectively, in *Rdh10+/−* male GM (Fig. 7*A*). *Cox5a* and *Cox8b* mRNA increased from 20 to 36% in *Rdh10+/−* female GM, but data were shy of statistical significance at a *p* ∼0.07. The mRNA amounts of other electron transfer chain components in *Rdh10+/−* GM did not differ from WT, indicating no decrease in total mitochondria (Fig. S11). These included *Ndufs2* (complex I), *Sdhb* (complex II), *Uqcrc2* (complex III), *Cycs* (cytochrome c), and *Apt5al* (complex V). Cox5a protein expression in GM lysates decreased in *Rdh10+/−* male GM by an average 28%, followed by a complex IV (cytochrome C oxidase) activity decrease of 13% and a fasting ATP decrease of 60% (Fig. 7, *B* and *C*). In contrast, Cox5a protein expression increased by 40% in *Rdh10+/−* female GM, accompanied by a 40% increase in complex IV activity, and >3-fold increase in fasting ATP (Fig. 7, *B* and *D*).

## Discussion

We have reported that eliminating one *Rdh10* copy *in vivo* (*Rdh10+/−*) reduced RA modestly in adult liver and white adipose (≤25%) and increased diet-induced obesity in both sexes (20). Only males endured liver steatosis when fed a HFD, and only females sustained increased bone marrow adipocyte formation, regardless of dietary fat. Here we report the impact of marginally reduced RA in muscle, showing an outcome likely influenced by increased estrogen in *Rdh10+/−* females, associated with an attenuated decrease in RA relative to males. These data provide further insight into RA modulation of estrogen effects and, reciprocally, estrogen modulation of RA effects. Assessing the whole body *Rdh10* heterozygote permitted assessment of overall Rdh10/RA impact on adiposity, insulin resistance, and carbohydrate *versus* fat use as fuels.

Male C57/Bl6 mice have larger body weights and more muscle mass compared with age-matched females (20). This difference in lean body mass, along with a greater reliance on fatty acid oxidation (RER), enables longer running times in WT males. The modest but significant decrease in *Rdh10+/−* male reliance on fatty acid oxidation and increased reliance on carbohydrates (RER), would affect running endurance. Decreases in male GM *Cpt1b* and *Hsl* mRNA would limit fatty-acid oxidation and prompt the increase in TAG. Decreases in *Gys* and *Glut1* would reduce glucose uptake and storage, contributing to whole-body glucose intolerance, as muscle accounts for >70% of insulin-stimulated glucose use (45). Decreases in *Pck1* and *G6pc3* would contribute to the decrease in *Rdh10+/−* male GM glucose. These decreases in muscle energy availability, verified by glucose intolerance and insulin resistance, plus impairment of complex IV amount and activity, would hinder ATP production in male GM and reduce energy for running endurance. Impaired energy use would prompt insulin resistance, which explains the decrease in *Fasn*, an insulin-regulated gene. The selective increase in type I (oxidative) fibers in *Rdh10+/−* males would not fully compensate for impaired endurance because the fibers included defective mitochondria.

The increased dependence on fatty acid oxidation in *Rdh10+/−* females (RER) and the increase in complex IV activity, with more efficient glucose uptake, produced more ATP and greater running endurance. These data show that RA has a complex impact on postnatal skeletal muscle function that alters ATP generation. These sexually dimorphic differences in muscle contribute to the whole-body phenotype of *Rdh10+/−* and very likely are influenced by sex hormones.

The surprising increase in female muscle performance with a decrease in RA seems to challenge concepts of RA function, because decreased RA usually diminishes cell, organ, and physiological performance (46). Then again, this outcome expands insight into interactions between RA and estrogen and is consistent with reports that estrogen enhances RA signaling, and its biosynthesis, whereas RA retards estrogen action. RA restricts estrogen receptor α action in the estrogen-sensitive human breast cancer cell line MCF7/BUS (47), inhibits estrogen-induced proliferation of MCF-7 cells (48), and promotes ERα degradation and reduces its transcriptional activity in breast cancer cells (49). RA promotes homeostatic maintenance of the mouse uterus, preventing estrogen-promoted proliferation (50, 51, 52). In contrast, estrogen sensitizes human breast cancer cells to RA by inducing RARβ (53). Estrogen also induces expression of stimulated by retinoic acid gene 8 (Stra8), showing that it can compensate for RA loss (54). Estrogen amplifies the effects of retinoids on ameliorating obesity in female mice (55). Estrogen enhances muscle mass and strength *in vivo* and promotes muscle regeneration (56, 57, 58). Notably, estrogen action in muscle increases insulin sensitivity, prevents lipid accumulation, and promotes metabolic health (59). Our cumulative data and these references support an estrogen contribution to improving the phenotype of *Rdh10*^*+/−*^ females, relative to males. In contrast to estrogen actions on RA signaling, androgens repress RAR mRNA in rat prostate (60), whereas RA suppresses androgen receptor expression in rat testis (61). The decreased performance by *Rdh10+/−* males could reflect a contribution of androgens to decreasing RA signaling and amplifying repression of RAR.

A multifaceted process controls skeletal muscle development, regeneration, and function (62). Ablating the myogenic transcription *Myog* after muscle development produces a mouse with enhanced performance in treadmill running, as a result of improved fuel metabolism (63), whereas ablating *Eif6* reduces running performance (34). The decreased running performance and restricted energy use of *Rdh10+/−* males reflect the increase and decrease, respectively, in expression of these genes. The juxtaposed increase in running performance and energy production of *Rdh10+/−* females reflects the decrease in *Myog* expression. Myogenic factors, Myf5 and MyoD commit stem cells to a skeletal muscle lineage. RA effects on Myog, Myf5, and MyoD have been studied during embryogenesis and in cell lines (64), but postnatal effects of endogenous RA have not received the same attention. Cumulative data in cell lines and chick limb buds indicate concentration-dependent RA effects on myogenesis (65, 66, 67). These results may reflect retinoid-related hormesis: *i.e.*, RA effects depend on concentrations. Beneficial effects occur as concentrations increase to an optimum, which presumably occurs at the low nM RA that occurs in tissues (68). As concentrations increase beyond an optimum, beneficial effects subside and pharmacological effects ensue; ultimately toxic effects prevail (69).

Hormesis also may contribute to sexual dimorphism. Estrogens *versus* androgens exert different effects on RA functions apparently by increasing or decreasing RAR expression, respectively, which would impact the RA dose–response curves based on sex. The sex-hormone/RA nexus would help explain how modest alterations in RA have contrary sex-related effects. Moreover, RA effects on myogenic differentiation depend on the exact cellular context, as location-specific interactions with the local chromatin environment affect transcriptional activity (70), as well as expression of distinctive retinoid-binding proteins (71).

## Conclusions

The present data complement insight into the antiobesity effects of RA by revealing that endogenous RA promotes skeletal muscle fuel use, mitochondrial function, and ATP biosynthesis in males, but restricts the same in females. These male and female specific actions also affect glucose tolerance and insulin resistance. Contrasting interactions between estrogens and androgens on RA biosynthesis and function suggest mechanisms for these dimorphic effects. This sexually dimorphic insight into RA in muscle contributes to understanding differences between males, premenopausal females, and postmenopausal females with respect to regulation of metabolic health. Given the complexity of RA actions *in vivo* and its hormesis-dependent effects, partial ablation of *Rdh10* should continue to provide opportunity to distinguish physiological from pharmacological and/or toxic actions of RA.

## Experimental procedures

### Mice and diets

Mouse experimental protocols were approved by the University of California-Berkeley Animal Care and Use Committee. *Rdh10*-floxed mice were bred with mice expressing CMV-Cre (B6.C-Tg (CMV-Cre) 1Cgn/J) to generate a whole-body knockout of *Rdh10* as described (20). Homozygous *Rdh10* knockout causes embryonic lethality, therefore *Rdh10* heterozygotes (*Rdh10+/−*) were studied. CMV-Cre+ littermates served as controls (WT). Mice were backcrossed into the C57BL/6J background >12 generations. Mice were fed an AIN93G purified diet containing 4 IU/g vitamin A with 50% calories as lard (HFD) since weaning (Research Diet, Inc, D17092103) and were fasted overnight prior to tissue collection to promote fatty acid use, unless noted otherwise. Mice were 4 to 5 months old. Female mice were synchronized in anestrous, because they were group-housed in the absence of males—the Lee–Boot effect (72, 73, 74)

### Respiratory exchange ratio (RER)

RERs were determined with a Columbus Instruments Comprehensive Laboratory Animal Monitoring System (CLAMS). Mice were acclimatized to chamber cages 24 h before values were recorded. The CLAMS was housed in a temperature controlled (23 °C), 12-h light/dark cycle room, with lights on at 7 AM. Mice had access *ad libitum* to food and water. Activities were measured simultaneously and showed no differences based on sex or genotype.

### Biochemical assays

ATP was quantified with an ATP Colorimetric/Fluorometric Assay Kit (Biovision K354). TAG concentrations were determined using a Triglyceride Colorimetric Assay Kit (Cayman 10010303). Glycogen contents were measured with a Glycogen Colorimetric/Fluorometric Assay Kit (Bio Vision K646). Complex IV activity was analyzed with a Complex IV Rodent Enzyme Activity Microplate Assay Kit (Abcam ab109911). Glucose was quantified with a Promega Kit (Glucose-Glo Assay).

### Run-to-exhaustion test

We used a motor-driven treadmill (Columbus Instruments, Exer-6M Open Treadmill) to assess running endurance. To encourage mice to run, tactile stimuli were provided by a manual light tap with a small paint brush to the hindquarters when the animal reached the back of the treadmill. On days 0 and 1, mice were placed on the treadmill to acclimate for 10 min with a speed of 10 m/min at 0% gradient. On day 2, running capacity was determined by placing mice on the treadmill without treadmill motion for 2 min for acclimation. Next, mice were subjected to a 5 min warm-up period at 10 m/min. Speed was then increased by 2 m/min each 2 min until a maximum of 20 m/min. Mice were then run to exhaustion, defined as sitting at the back wall for 10 s despite continual tapping with the brush. Work (Joules) was calculated from body weight (kg) × 9.8 × distance run in m.

### Antibodies

Primary antibodies: Myh2 (3 μg/ml, Developmental Studies Hybridoma Bank, sc-71, AB_2147165), Myh4 (3 μg/ml, Developmental Studies Hybridoma Bank, BF-F3, AB_2266724), Myh7 (3 μg/ml, Developmental Studies Hybridoma Bank, BA-F8, AB_10572253), Desmin (1:20. Thermo Fisher Scientific, MA5-13259), Cox5a (1:1000, Abcam, ab110262, AB_10861723), Glut1 (1:200, Abcam, ab652, AB_305540), Myf5 (1:10,000, Abcam, ab125078, AB_10975611), Fasn (1:1000, Abcam, ab22759, AB_732316). Secondary antibodies: goat-anti-mouse IgG2b Alexa 350 (1:200, Thermo Fisher A-21140, AB_2535777), goat-anti-mouse IgG1 Alexa 488 (1:200, Thermo Fisher A-21121, AB_2535764), goat-anti-mouse IgM Alexa 555 (1:200, Thermo Fisher A-21426, AB_2535847), IRDye 800CW goat anti-mouse IgG Secondary Antibody (1:10,000, Licor, 926-32210, AB_621842), IRDye 800CW goat anti-rabbit IgG Secondary Antibody (1:10,000, Licor, 926-32211, AB_621843), Anti-rabbit IgG HRP-linked Antibody (1:200, Cell Signaling Technology, 7074P, AB_2099233).

### Histology and immunofluorescence

Entire GM were dissected from legs, snap-frozen in liquid nitrogen, and stored at −80 °C. Cryosections (10 μm each) were harvested from frozen muscles with a Cryostat (Leica CM3050) and processed for histological and immunofluorescence analysis. Sections were stained with hematoxylin (Sigma GHS3) and eosin Y (RICCA 2845-32), washed with distilled water, and mounted in Shur Mount (General Data LC-W). For CSA quantification, all fibers in each field were quantified from four mice per group with four sections for each mouse, except for one of the female WT and one female Het for which three sections were analyzed. In total 1454 to 1635 fiber sections were quantified for CSA. Oil red O was used to stain lipids (75). Sections were mounted in Shur Mount and glycogen was visualized by Periodic Acid-Schiff staining (PAS) (MilliporeSigma 395B-1KT) following manufacturer’s instructions. Slides were imaged with a Zeiss AxioObserverZ1 microscope at 40× magnification, using QImaging 5MPx a MicroPublisher color camera (Biological Imaging Facility, UC-Berkeley).

For immunofluorescence imaging, sections were warmed to room temperature for 40 min and washed three times with 0.1% PBST. After blocking with 5% NGS/PBST (normal goat serum in 1% Tween 20/phosphate-buffer) 1 h at room temperature, sections were incubated in 5% NGS/PBST with a mixture of three primary antibodies against Myh7 (IgG2b), Myh2 (IgG1), and Myh4 (IgM), obtained from DSHB at the University of Iowa. After three 5 min washes in PBST, sections were incubated 1 h at room temperature with a mixture of three goat anti-mouse secondary antibodies against IgG2b (Alexa 350), IgG1 (Alexa 488), and IgM (Alexa 555) in 5% NGS/PBST, followed by three washes in PBST. For analysis of centralized nuclei, sections were first incubated in the primary anti-Desmin monoclonal antibody overnight at 4 °C. For nuclear staining, sections were incubated in 300 nM DAPI (4′6-diamidino-2-phenylindole, Thermo Fisher Scientific, D1306) working solution for 5 min at room temperature, followed by washing in PBS. Sections were mounted with Prolong Diamond Anti-fade mountant (Thermo Fisher P36965) and sealed with colorless nail polish. Washing and blocking for IHC staining were the same as for immunofluorescence. Slides were incubated overnight at 4 °C with primary glucose transporter 1 antibodies (Glut1), washed with 0.1% PBST, and incubated with goat-anti-rabbit IgG HRP. Stained slides were examined at 40× magnification with oil immersion using Zeiss Axiovert M1 fluorescent microscope equipped with Hamamatsu Orca CCD camera and CoolLED pE-300 white LED illuminator, and Zeiss Z1 AxioObserver inverted microscope equipped with a 40× immersion lens, X-Cite 120 LED fluorescence source and Qimaging Retiga SRV CCD camera (Biological Imaging Facility, UC Berkeley). To quantify Glut1 intensity, the reciprocal of brightness in the blue channel-measure was used in Image J.

### RNA extraction and qPCR

Total RNA was extracted from tissues with TRIzol reagent (Life 15596026). The RNA concentration was measured with a Nanodrop spectrophotometer and reverse-transcribed with iScript (Bio-Rad 1708841), followed by qPCR reactions using Prime Time Gene Expression Master Mix (Integrated DNA Technologies 1055771). qPCR data were normalized to the housekeeping genes *Gusb* and *Tbp.* Each *Rdh10+/−* mRNA was normalized to its WT littermates. We used predesigned primers purchased from Integrated DNA technology. Primer details are provided in Supporting information (Table S1).

### Western blots

Immunoblotting was performed as described (76). Muscles were thawed on ice and homogenized with a TissueLyser II (Qiagen Inc) in 1 ml cold RIPA buffer (Thermo Fisher 89900) supplemented with protease inhibitor (Thermo Fisher A32965) and phosphatase inhibitor (Roche 4906845001). Tissue lysates were denatured and centrifuged. Protein was quantified in the supernatant with the BCA protein assay (Thermo Fisher 23225), subjected to SDS-PAGE, and transferred onto PVDF membranes (Bio-Rad 1620177). Membranes were blocked with 5% fat-free milk, incubated with primary antibodies overnight (4 °C), and with secondary antibodies 1 h at room temperature. Membranes were scanned and signal intensities were quantified using a LiCOR Odyssey imager. Protein loaded per lane was determined with Coomassie blue staining (Thermo Fisher 46-5034) and analyzed with NIH Image J. Data were normalized to total protein loaded per lane. Units are arbitrary (AU), normalized to WT males set to 1, unless noted otherwise.

### RA quantification

RA was quantified by LC/MS/MS as published, except methanol was used instead of ethanol, and homogenates were centrifuged at 1200*g* for 5 min to remove particulates (77). LC was done as published (78).

### Statistics

All data are present in this manuscript. Data were plotted as means ± SD, unless noted otherwise. Two-tailed unpaired t-tests were used to analyze results between two groups. Welch’s corrections were applied to groups without equivalent SD. Significantly different from WT: ∗*p* < 0.05, ∗∗ *p* < 0.01, ∗∗∗*p* < 0.001, unless noted otherwise; n = number of mice per genotype/sex.

## Data availability

All data are present in this manuscript.

## Supporting information

This article contains supporting information.

## Conflict of interest

The authors declare that they have no conflicts of interest with the contents of this article.
