# Supplementary material for: Retinoic acid exerts sexually dimorphic effects on muscle energy metabolism and function
Source: J Biol Chem. 2021 Aug 19;297(3):101101. doi: 10.1016/j.jbc.2021.101101 (PMC8441203; doi:10.1016/j.jbc.2021.101101)
Supplement: Supplemental Table S1 [file mmc1.pdf]

Supporting Table 1. Primers used for qPCR.

Gene Symbol	Assay ID	Ref Sequence		Gene Symbol	Assay ID	Ref Sequence
Gusb	Mm.PT.39a.22214848	NM_010368(1)		*Mcad*	Mm.PT.58.9316361	NM_007662(1)
Adcy5	Mm.PT.58.37585706	NM_001012765(1)		*Myf5*	Mm.PT.58.5271235	NM_008656(1)
Adrb2	Mm.PT.58.29310038.g	NM_007420(1)		*Myh2*	Mm.PT.58.13701815	NM_001039545(1)
Atgl	Mm.PT.56a.13182140	NR_028142(1)		*Myh4*	Mm.PT.58.29699487	NM_010855(1)
Atp5a1	Mm.PT.58.6486863	NM_007505(1)		*Myh7*	Mm.PT.58.17465550.g	NM_080728(1)
Cd36	Mm.PT.58.32162630	NM_001159558(1)		*Myog*	Mm.PT.58.6732917	NM_031189(1)
Cox5a	Mm.PT.58.13914094	NM_007747(1)		*Myoz1*	Mm.PT.58.9542275	NM_021508(1)
Cox8b	Mm.PT.58.32066295	NM_007751(1)		*Ndufs2*	Mm.PT.58.33517076	NM_153064(1)
Cpt1	Mm.PT.58.10147164	NM_013495(1)		*Pfkm*	Mm.PT.58.11040737	NM_001163487(3)
Cpt1b	Mm.PT.58.23409278	NM_009948(1)		*Ppara*	Mm.PT.58.9374886	NM_001113418(2)
Cycs	Mm.PT.58.28767188	NM_007808(1)		*Ppard*	Mm.PT.58.6994542	NM_011145(1)
Cyp26b1	Mm.PT.56a.12507788	NM_001177713(2)		*Pten*	Mm.PT.56a.8966497	NM_008960(1)
Dhrs7c	Mm.PT.58.6664141	NM_001013013(1)		*Pygm*	Mm.PT.58.10876047	NM_011224(1)
Dhrs9	Mm.PT.58.12333368	NM_175512(1)		*Raldh1*	Mm.PT.58.30016484	NM_013467(1)
Eif6	Mm.PT.58.12695972	NM_010579(1)		*Raldh2*	Mm.PT.58.12196815	NM_009022(1)
Fasn	Mm.PT.58.14276063	NM_007988(1)		*Raldh3*	Mm.PT.58.11310697	NM_053080(1)
Glut1	Mm.PT.58.7590689	NM_011400(1)		*Rarb*	Mm.PT.58.32130670	NM_011243(1)
Glut4	Mm.PT.58.9683859	NM_009204(1)		*Rdh10*	Mm.PT.58.9541215	NM_133832(1)
Gys1	Mm.PT.58.30132192	NM_030678(1)		*Sdhb*	Mm.PT.58.42320868	NM_023374(1)
Hif1a	Mm.PT.58.11211292	NM_010431(1)		*Tbp*	Mm.PT.39a.22214839	NM_013684(1)
Hk2	Mm.PT.58.32698746	NM_013820(1)		*Uchl1*	Mm.PT.58.32038186	NM_011670(1)
HSL	Mm.PT.58.6342082	NM_010719(1)		*Uqcrc2*	Mm.PT.56a.16136852	NM_025899(1)
