# Supplementary material for: Retinoic acid exerts sexually dimorphic effects on muscle energy metabolism and function
Source: J Biol Chem. 2021 Aug 19;297(3):101101. doi: 10.1016/j.jbc.2021.101101 (PMC8441203; doi:10.1016/j.jbc.2021.101101)
Supplement: Supplemental Figures S1–S11 [file mmc2.pdf]

Supporting Information

Retinoic acid exerts sexually dimorphic effects over muscle energy metabolism and function

Yaxin Zhao[1], Marta Vuckovic[1], Hong Sik Yoo[1], Nina Fox[1], Adrienne Rodriguez[1], Kyler McKessy[1], and Joseph L. Napoli[1,2]

[1]Department of Nutritional Sciences and Toxicology
Graduate Program in Metabolic Biology
The University of California-Berkeley
Berkeley CA 94720, USA

[2]Address correspondence to:
J. Napoli
119 Morgan Hall
UC-Berkeley
Berkeley CA, USA 94720
jna@berkeley.edu

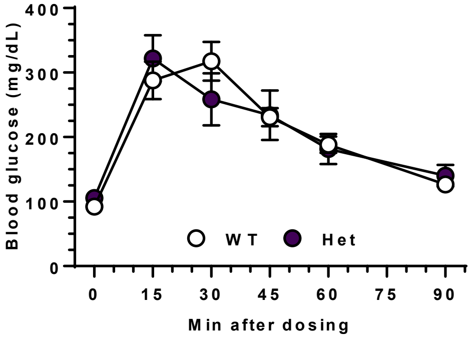

Figure S1. GTT of females. Mice were fasted 16 h and then dosed i.p. with 1.5 g glucose/kg body weight. Six to 8 mice/genotype. Blood glucose from tail tips was measured with a glucometer.

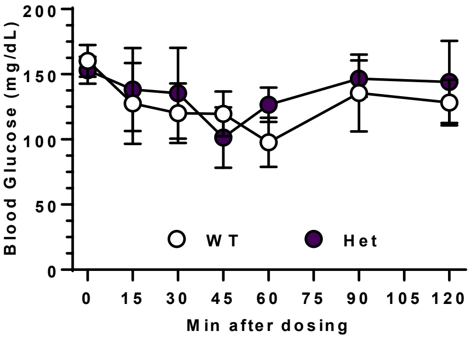

Figure S2. ITT of females. Mice were fasted 4 h and placed in individual cages without food, but with free access to water. Insulin (0.5 IU/kg body weight) was injected i.p. into 4 to 5 mice/genotype. Blood glucose from tail tips was measured with a glucometer.

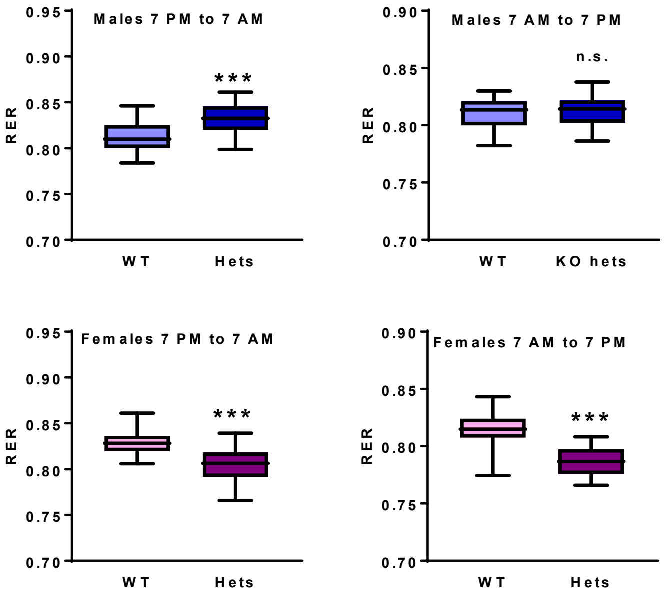

Figure S3. RER averages. RER (CO_2/O_2) during ambient temperature and ad lib feeding a high-fat diet. Data are means +/- SEM (n = 6 to 10 mice/genotype/sex. ***P < 0.0001). ns, not significant.

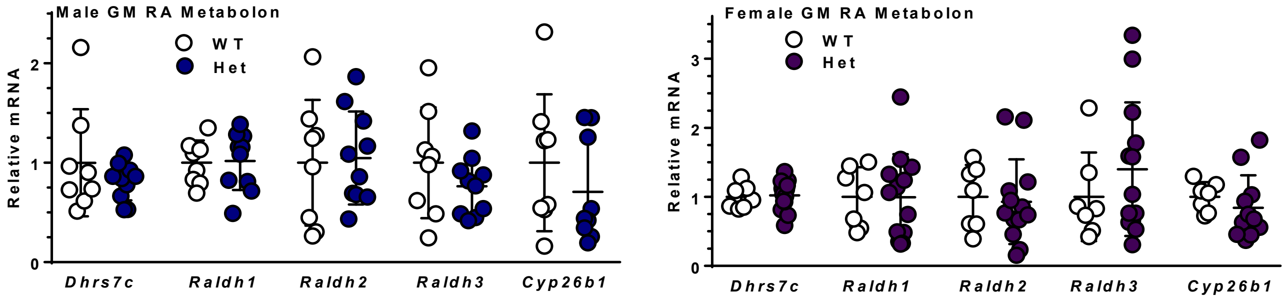

Figure S4. mRNA of retinoid metabolic genes not changed in Het. Seven to 13 mice per gene/genotype/sex. Muscle does not express *Rdh1* mRNA.

Figure S5. qPCR of *Myod* mRNA. Four to 10 mice per genotype/sex.

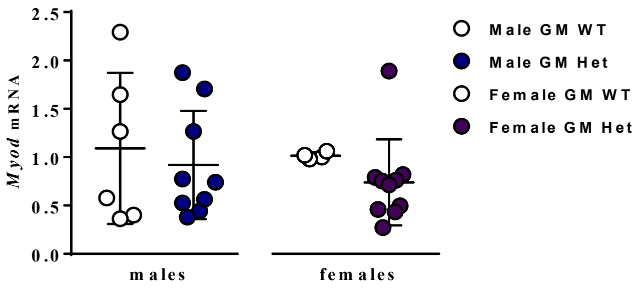

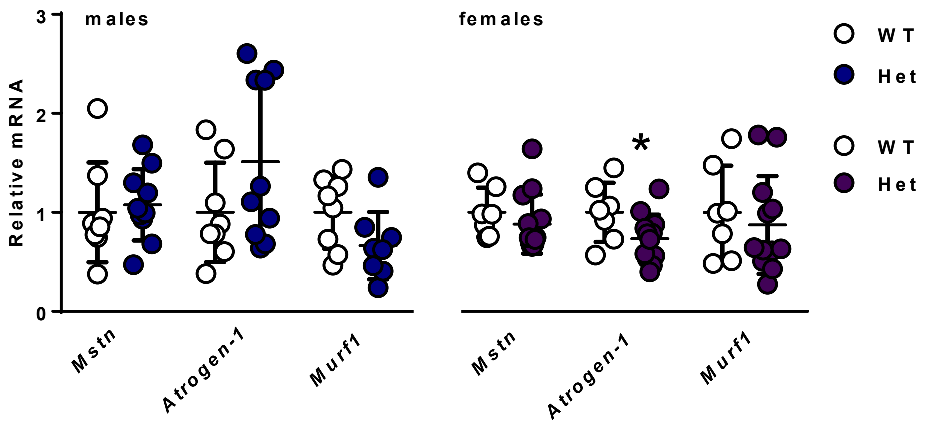

Figure S6. mRNA of genes associated with skeletal muscle mass and function. Seven to 13 mice gene/geneotype/sex.

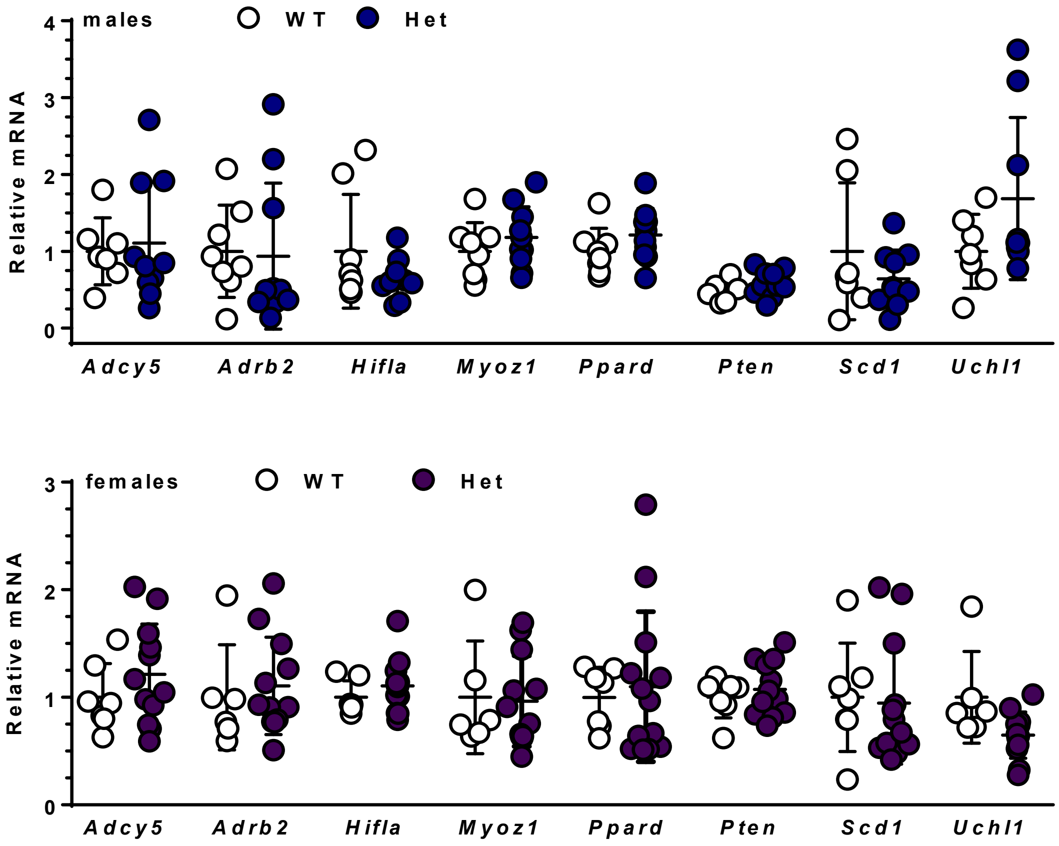

Figure S7. mRNA of genes that affect running endurance in mice. Four to 10 mice per gene/genotype/sex.

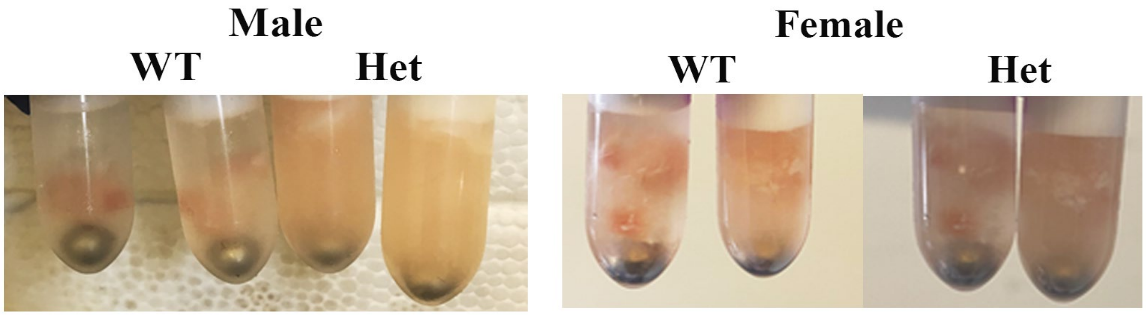

Figure S8. Representative images of GM muscle homogenization. Entire muscles were homogenized with Tissuelyser II (Qiagen) at 30 Hz for 15 min. WT of both sexes remained largely intact, whereas Het of both sexes were substantially disrupted under the same conditions.

Figure S9. Total protein amounts in GM. Six to 8 mice per muscle/genotype/sex.

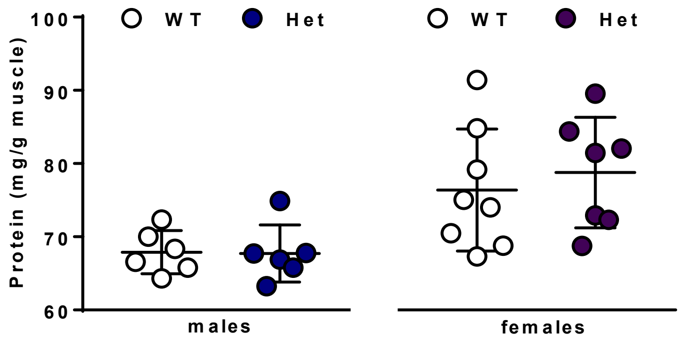

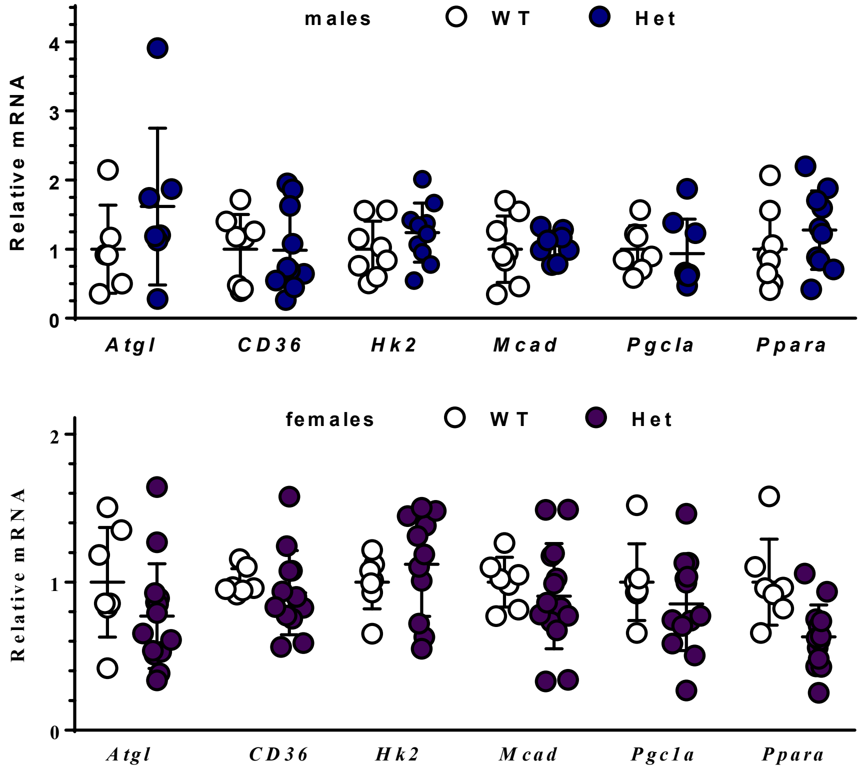

Figure S10. mRNA of fatty acid metabolism genes in GM. Six to 14 mice per gene/genotype/sex.

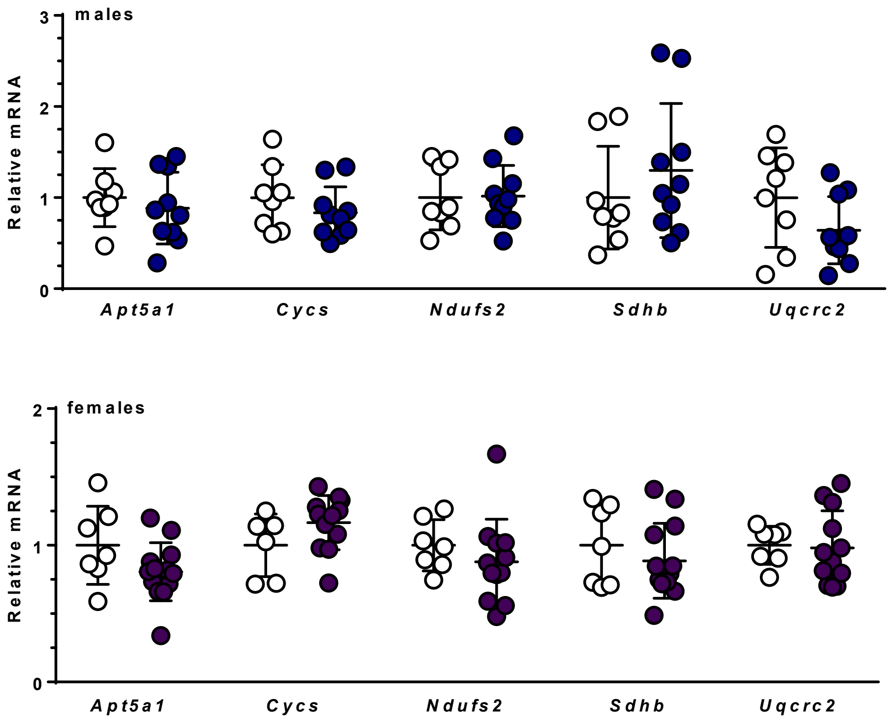

Figure S11. Mitochondria function genes. Six to 12 mice per gene per genotype per sex/muscle.
